# Association between perinatal depressive symptoms and parental cohabitation status in a Nordic high-income country

**DOI:** 10.1371/journal.pone.0341051

**Published:** 2026-02-12

**Authors:** Iliana Liakea, Natasa Kollia, Malva Nord, Emma Fransson, Maja Bodin, John L. Cox, Emma Bränn, Alkistis Skalkidou

**Affiliations:** 1 Department of Women’s and Children’s Health, Uppsala University, Uppsala, Sweden; 2 Behavioral Science Institute, Radboud University, Nijmegen, Netherlands; 3 Department of Nutrition Dietetics, School of Health Science and Education, Harokopio University, Athens, Greece; 4 Department of Microbiology, Tumor and Cell Biology, Karolinska Institutet, Stockholm, Sweden; 5 The National Centre for Knowledge on Men’s Violence Against Women, Uppsala University, Uppsala, Sweden; 6 Keele University, Keele, United Kingdom; 7 Institute of Environmental Medicine, Karolinska Institutet, Stockholm, Sweden; 8 Center for Epidemiology and Community Medicine, Region Stockholm, Stockholm, Sweden; Group for Technical Assistance / Asian College for Advance Studies, Purbanchal University, NEPAL

## Abstract

Change in cohabitation status could influence the economic security and well-being of parents and their children. However, literature concerning the association between the duration of perinatal depression (PND) exposure and change in parental cohabitation status is limited. Therefore, this study aimed to assess whether the presence and persistence of maternal PND symptoms are associated with changes in parental cohabitation status up to six years postpartum. Using data from 4,344 persons in the Swedish BASIC and U-BIRTH cohort studies, maternal depressive symptoms were assessed at three time points (during pregnancy, 6 weeks, and 6 months postpartum) using the Edinburgh Postnatal Depression Scale. Cohabitation status was measured at 6 weeks and 6 years postpartum. Logistic regressions estimated odds ratios (ORs) for non-cohabitation associated with the number of PND-positive time points. Mothers with positive screenings at more than one time point for depressive symptoms had higher odds of not cohabiting at both 6 weeks and 6 years postpartum. At 6 years, mothers with depressive symptoms at all three time points had over four times the odds of not cohabiting (OR 4.1, 95% CI 1.7–9.5). However, most associations lost significance after full adjustment for sociodemographic and psychosocial factors, except the association between prolonged PND (3 positive screenings) and non-cohabitation at six years postpartum. Prolonged PND symptoms may increase the risk of long-term parental separation. Although confounding factors reduce the strength of this association, findings underscore the need for extended mental health monitoring and support for perinatal persons.

## Introduction

During the 21^st^ century, the share of children with separated parents in Sweden steadily increased [1]. In 2011, 18.5% of individuals under 24 years old in Sweden had experienced parental separation or divorce, with this percentage rising to 19.8% by 2023 [1]. Some factors associated with separation are lower education, unemployment, lower marital and life satisfaction, parental strain, stress, alcohol or drug abuse, infidelity, incompatibility, personality disorders, physical or mental abuse, and psychiatric morbidity [2–5]. While Sweden has a dual income and dual caretaker system that partly supports the adults after a separation, globally, divorce or separation influences adults’ economic security and well-being [6]. In Sweden, this system helps ensure that both parents remain actively involved in their child’s upbringing through joint custody laws, generous parental leave, subsidized childcare, and flexible work policies, fostering financial independence and shared caregiving responsibilities [7]. Economic consequences, though, such as loss of income, especially for women divorcees [8], are associated with mood disorders and substance [9] and alcohol abuse [10]. Although studies suggest that women often initiate the process of separation [2,5], previous studies find single mothers more likely to experience economic hardship, mental health or affective disorders, and mental distress [11–13].

Today, separation is understood as a more complex process rather than a single, one-time event. Particularly, former partners may decide to cohabitate after their separation due to practical reasons, such as not changing children’s school and habits, and/or financial motives [14]. In fact, for some parents the decision to cohabit after separation could be regarded as an “ideological” gesture, as it is perceived by parents as the most advantageous method for sustaining the parental partnership and mitigating potential negative repercussions for their children [14]. Hence, the examination of cohabitation may be particularly pertinent in the contemporary era.

In parallel, perinatal depression (PND) is a major public health issue that affects more than 10% of the mothers [15] and may impinge on the mother’s life and their surroundings, including their family and society. Mothers who report depressive symptoms postpartum rate their relationship with both their child and partner as being less close, warm, confident, and easy over the first year postpartum compared to non-depressed [16]. They also report feelings of loneliness in parenthood, and assuming the whole responsibility for the household, while they rate emotional closeness and intimacy as being low in their relationship with their partner [17]. In parallel, there appears to be an association between sexual activity and postpartum depression, where mothers with depression demonstrate a propensity to delay the resumption of sexual activity after the delivery [18]. Furthermore, maternal PND often co-occurs with paternal postpartum depression [19,20].

Lack of intimacy, insufficient communication, and impaired dyadic consensus seem related to both PND [16–18] and parental separation [3]. Dyadic consensus, a crucial component of marital satisfaction, refers to the extent to which partners collaboratively engage in decision-making and reach mutually agreed-upon resolutions within their shared life. It encompasses various domains including cohesion, satisfaction, consensus, and affective expression within the partnership [21,22]. PND has been negatively associated with couple’s satisfaction [23] and has been positively related with the deterioration in intimate and sexual activity [24] which in turn are related to higher levels of parental separation [3]. Additionally, separated mothers and fathers report lower levels of overall dyadic consensus and higher levels of depressive symptoms than their non-separated counterparts [25]. However, the literature assessing directly the association between PND and parental separation is, to our knowledge, limited. Kerstis et al. [25] explored the association between depression three months after childbirth and parental separation at 6–8 years after childbirth and found that lowered dyadic consensus, depressive symptoms, and parental stress increased the likelihood of parental separation. Widarsson et al. [5] assessed parental stress and depressive symptoms at three months postpartum in correlation to parental separation 9–11 years after childbirth and found that separated parents had higher Edinburgh Postnatal Depression Scale (EPDS) [26] scores than non-separated. The studies by Widarsson et al. [5] and Kerstis et al. [25] both assessed depression only at 3 months postpartum. To our knowledge, no study has examined PND at multiple time points in relation to parental separation, despite evidence that prolonged PND has cumulative adverse effects on mothers and their families [27]. Since relationship challenges may evolve over time, evaluating depressive symptoms at multiple time points can provide valuable insight into how prolonged exposure to these symptoms affects relationship difficulties.

We chose depressive symptoms as the exposure variable because evidence suggests that relationship quality often declines after childbirth, especially for parents experiencing postpartum mental health challenges [28]. These parents are more likely to report lower levels of partner support which is recognized as a main risk factor along with other relationship measures, such as relationship satisfaction and dyadic consensus, for parental separation [3]. Therefore, this study aimed to assess whether the presence of maternal depressive symptoms at multiple time points (during pregnancy, six weeks, and six months postpartum) increases the likelihood of parental separation at six weeks and six years postpartum, with a particular focus on parental cohabitation. To achieve this, we utilized a study design incorporating multiple time points, where maternal depressive symptoms were assessed as the exposure and parental cohabitation served as the outcome.

## Materials and methods

### Study population

This study was conducted within the framework of the BASIC study (Biology, Affect, Stress, Imaging and Cognition) [29] and the ongoing U-BIRTH study [30]. BASIC was a population-based, prospective cohort study, in the county of Uppsala, Sweden, recruiting between September 1^st^, 2009 and November 31^st^, 2018 (n = 6,474 pregnancies). The focal point of BASIC was to examine biological and sociopsychological risk factors, comorbidities, and consequences of PND, and thus participants were followed from pregnancy up to 12 months postpartum. Since 2012, individuals who participated in the BASIC study have been invited to participate in the U-BIRTH study [30] (initiated April 4^th^, 2012 and data collection is still ongoing), which is a follow-up study starting from when the child is 18 months old and continues until the age of 11 years, collecting data on both the mother and the child (n = 1,129 at time of data analysis).

Recruitment in the BASIC study occurred during the 16^th^-18^th^ gestational week when persons had scheduled their routine ultrasound at Uppsala University Hospital. In parallel, persons who were scheduled for an elective cesarean section at Uppsala University Hospital were recruited while attending their pre-operative consultation. After signing the written consent form, recruited persons completed, among others, the Edinburgh Postnatal Depression Scale (EPDS) at baseline, at gestational week 32, as well as at 6 weeks, and 6 months postpartum. Furthermore, data concerning maternal health was collected through linkage to clinical records. Participants included in U-BIRTH received an additional survey at 6 years postpartum.

**Fig 1:**
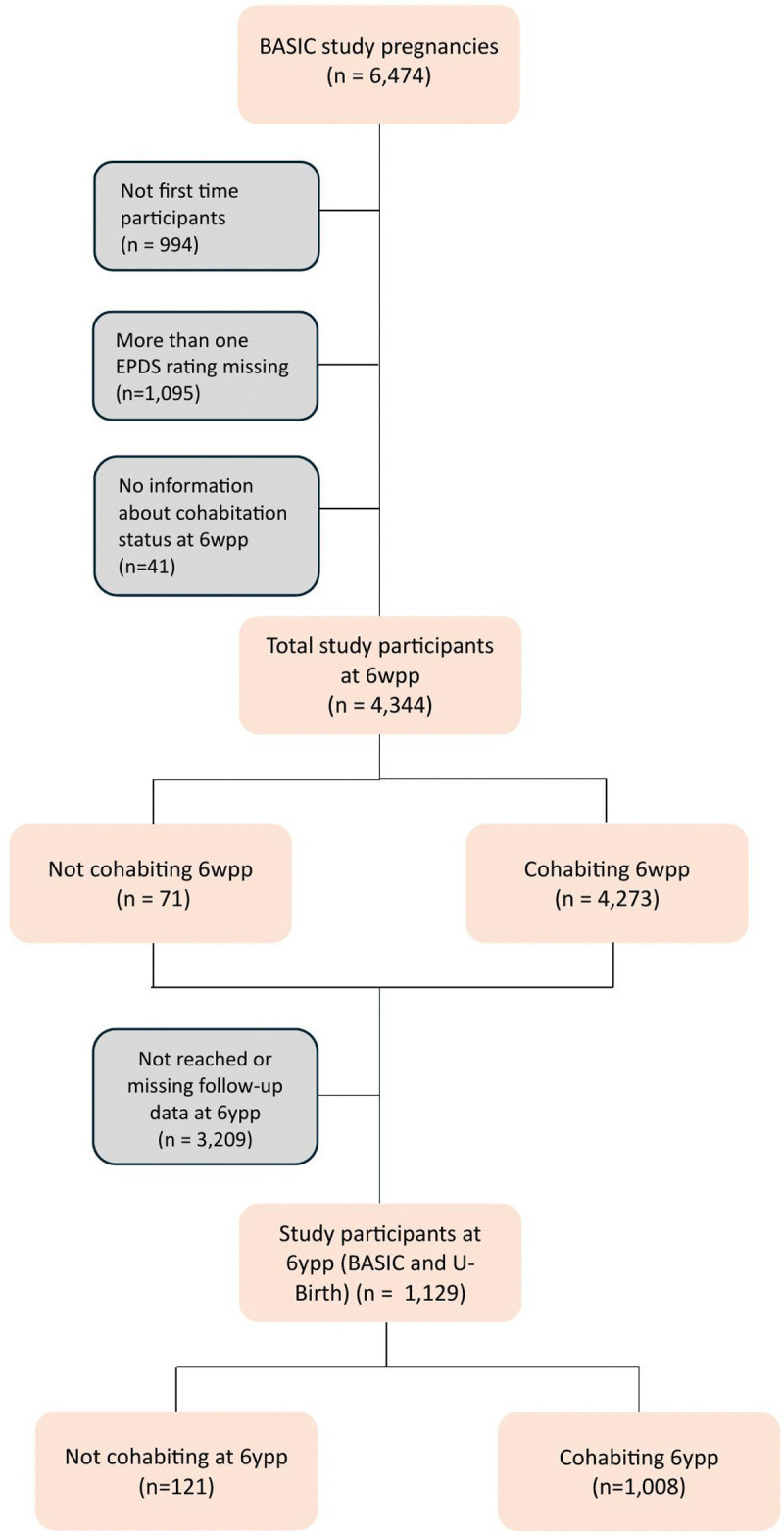
Flowchart portraying the exclusion criteria of the study. Grey boxes represent exclusion. 6wpp = 6 weeks postpartum 6ypp = 6 years postpartum.

This study included persons who had participated in the BASIC and the U-BIRTH study. The data were accessed for research purposes on February 24, 2022. Information that could identify individual participants was available during or after data collection. If a mother had participated in more than one pregnancy, only the first pregnancy was included. The flowchart (**Fig 1**) depicts the number of participants (mothers) in each analysis. Importantly, the study’s analysis of the 6-year postpartum data only included mothers who had been invited to complete the questionnaire at that point, as not all mothers included in the study at the 6-week postpartum stage had reached the 6-year postpartum stage at the time of analysis.

### Outcome

In this study, parental separation was operationally defined as cohabiting or not cohabiting with the child’s other parent, due to methodological constraints. Alteration in cohabitation status to denote a transition from co-residence to living apart is not entirely equivalent to divorce or parental separation. However, this particular facet of separation is the focal point of interest for the current study. The outcome, change in parental cohabitation status, was assessed at two different time points: at 6 weeks postpartum, and 6 years postpartum. At 6 weeks postpartum, the participants responded to the question “Is your partner free from work?”. Those who answered, “I do not live with my partner”, were categorized as Not cohabiting at 6 weeks postpartum, whereas mothers who chose any of the other response options (“yes” or “no”) were categorized as Cohabiting at 6 weeks postpartum. Notably, some mothers could have been non-cohabiting right from the commencement of their pregnancy, owing to factors such as living apart, imprisonment, death, or being single mothers who conceived through assisted reproductive technology. For the mothers who had missing data on this question, information on the latest clinical record entry (from during pregnancy) which mentioned marital/cohabitation status was extracted and examined. Persons listed as “single” were categorized as Not cohabiting at 6 weeks postpartum, whereas mothers listed as “cohabiting and/or married” were categorized as Cohabiting at 6 weeks postpartum.

At 6 years postpartum, the participants answered the question “Are you living together with the other parent of the child?”. Mothers replying “yes” were categorized as Cohabiting at 6 years postpartum whereas mothers replying “no” were categorized as Not cohabiting at 6 years postpartum.

### Exposure

The presence and persistence (i.e., the number of positive EPDS screenings) of PND symptoms were considered the exposure and were based on the number of time points with positive PND symptom screenings, measured by the EPDS. EPDS is a 10-item self-assessed screening tool for PND, where each item is responded to upon a Likert scale from 0–3 with higher scores indicating more severe symptoms, giving a maximum score of 30. Notably, the EPDS is a screening tool for depressive symptoms and not a diagnostic tool, hence, only PND symptoms were assessed in this study, and not clinical depressions status. A PND symptom screening was considered positive if the EPDS score was ≥ 13 in any survey completed during pregnancy [31] and/or ≥12 at 6 weeks postpartum or 6 months postpartum [32]. Participants with more than one missing EPDS screening from either the antenatal assessments, 6 weeks postpartum, or 6 months postpartum surveys were excluded.

For the analysis, at 6 weeks postpartum, mothers were categorized as (1) not having depressive symptoms if the antenatal and the 6 weeks postpartum screenings were both negative, (2) having depressive symptoms with one positive screening, if either antenatal or at 6 weeks postpartum were positive, or (3) having depressive symptoms with two positive screenings if both antenatal and 6 weeks postpartum were positive.

For the analysis at 6 years postpartum, mothers were categorized as (1) not having depressive symptoms if at least two of the antenatal, 6 weeks postpartum, and 6 months postpartum screenings were negative and none were positive, (2) having one depressive symptom screening if either antenatal, 6 weeks postpartum, or 6 months postpartum screening were positive, (3) having two depressive symptom screenings if at least two screenings were positive, and (4) having three depressive symptom screenings if all three screenings (the antenatal, 6 weeks postpartum, and the 6 months postpartum screening) were positive.

### Covariates

Age at childbirth was categorized into three groups using the 25th and 75th percentiles as cut-offs. Educational level was classified as university attendance (yes/no), and parity as primiparous or multiparous. Further, self-reported history of depression defined as a previous depressive episode or contact with a psychiatrist, psychologist, or mental health counselor (yes/no), and having a planned pregnancy (yes/no) were also considered as possible confounders. Additionally, exposure to stressful life events during the past year, measured at 6 weeks postpartum, was considered a possible confounder. The events covered interpersonal loss (e.g., the death of a close friend or relative), personal financial problems, relocation, and serious illness or injury [33]. The number of events was summarized and categorized as 0–1 stressful life events and ≥2 stressful life events. Lastly, mothers reported if they had experienced violence in previous and/or current relationships (yes/no).

### Statistical analyses

All analyses were performed using Statistical Package for the Social Sciences (SPSS) Statistics build 1.0.0.1508 (64-bit edition). Results with p-values <0.05 were considered statistically significant, and confidence intervals (CI) were set to 95%. Primary univariate analyses were conducted to assess the covariates in relation to the outcome, i.e., parental cohabitation (at 6 weeks and 6 years postpartum).

To assess the association between PND and parental cohabitation at 6 weeks and 6 years postpartum, we conducted multivariate logistic regressions (F(x) = 1/(1 + e^-(b0 + bx1+bx2+…^)). The first model (M0) was unadjusted. In Model 1 we adjusted for age, level of education, and parity at 6 weeks postpartum. In the final model, Model 2, we additionally adjusted for history of depression, planned pregnancy, and stressful life events or experiences of violence.

To avoid possible misclassification of cohabitation, we repeated the analysis of cohabitation status at 6 years postpartum, but then excluded mothers who were already not cohabiting at 6 weeks postpartum.

### Ethical approval

When invited to participate in the BASIC study and the U-BIRTH study, all participants received written information concerning the study, and thereafter submitted their written consent. Consent was given separately for each modality the participant wished to participate in. Both the BASIC and U-BIRTH studies have been approved by the Regional Ethical Review Board in Uppsala (BASIC: Dnr 2009/171, U-BIRTH: Dnr 2012/010, both with amendments), and all methods were performed following the relevant guidelines and regulations.

## Results

In this study, 71 (1.6%) of the 4,344 included participants reported not cohabiting with their partner at 6 weeks postpartum. Non-cohabiting mothers at 6 weeks postpartum were more likely to have experienced prior depression, unplanned pregnancies, stressful life events, and violence in relationships compared to mothers cohabiting at 6 weeks postpartum. Further, non-cohabiting mothers at 6 weeks postpartum were more likely older, and on sick leave or unemployed compared to cohabiting mothers (**Table 1**).

**Table 1:** Background characteristics of women who were cohabiting and non-cohabiting at 6 weeks and 6 years postpartum.

	Family status at 6wpp		Family status at 6ypp		Family status at 6ypp excluding women not cohabiting at 6weeks	
	Cohabitating	Non-cohabiting	Cohabitating	Non-cohabiting	Cohabitating	Non-cohabiting
	n (%)	n (%)	n (%)	n (%)	n (%)	n (%)
n	4,273	71	1,008	121	1,007	112
Age group (during delivery)						
≤ 27 yrs	850 (20%)	12 (17%)	169 (17%)	22 (18%)	169 (17%)	21 (19%)
28–34 yrs	2,403 (56%)	29 (41%)	581 (58%)	61 (50%)	581 (58%)	58 (52%)
≥ 35 yrs	1,019 (24%)	30 (42%)	258 (25%)	38 (32%)	257 (26%)	33 (29%)
Unknown	1 (0%)	0 (0%)	0 (0%)	0 (0%)	0 (0%)	0 (0%)
Level of education						
Lower	927 (22%)	22 (31%)	142 (14%)	33 (27%)	142 (14%)	32 (29%)
University	3,138 (73%)	46 (65%)	824 (82%)	82 (68%)	823 (82%)	75 (67%)
Unknown	208 (5%)	3 (4%)	42 (4%)	6 (5%)	42 (4%)	5 (4%)
Parity						
Primipara	2,299 (54%)	48 (68%)	509 (50%)	65 (54%)	508 (50%)	58 (52%)
Multipara	1,806 (42%)	21 (30%)	491 (49%)	55 (45%)	491 (49%)	53 (47%)
Unknown	168 (4%)	2 (3%)	8 (1%)	1 (1%)	8 (1%)	1 (1%)
Planned pregnancy						
No	587 (14%)	27 (38%)	118 (12%)	22 (18%)	118 (12%)	19 (17%)
Yes	3,459 (81%)	41 (58%)	839 (83%)	92 (76%)	838 (83%)	87 (78%)
Unknown	227 (5%)	3 (4%)	51 (5%)	7 (6%)	51 (5%)	6 (5%)
History of depression or previous contact with psychiatrist/psychologist/counselor						
No	1,796 (42%)	13 (18%)	485 (48%)	32 (26%)	485 (48%)	30 (27%)
Yes	2,288 (54%)	55 (77%)	481 (48%)	84 (70%)	480 (48%)	78 (70%)
Unknown	189 (4%)	3 (4%)	42 (4%)	5 (4%)	42 (4%)	4 (4%)
Number of stressful life events (during the year prior to delivery)						
0-2	3,704 (87%)	54 (76%)	901 (89%)	101 (83%)	900 (89%)	93 (83%)
3-10	507 (12%)	16 (23%)	103 (10%)	19 (16%)	103 (10%)	18 (16%)
Unknown	62 (1%)	1 (1%)	4 (1%)	1 (1%)	4 (0%)	1 (1%)
Experienced violence in relationships (either in current or previous)						
No	3,520 (82%)	48 (68%)	867 (86%)	81 (67%)	866 (86%)	75 (67%)
Yes	454 (11%)	17 (24%)	73 (7%)	33 (27%)	73 (7%)	32 (29%)
Unknown	299 (7%)	6 (8%)	68 (7%)	7 (6%)	68 (7%)	5 (4%)

6wpp = six weeks postpartum, 6ypp = six years postpartum.

At 6 years postpartum, the number of mothers not cohabiting had increased to 121 (10.7%). Non-cohabiting mothers at 6 years postpartum were more likely to have experienced prior depression, and violence in relationships. Further, they had lower education, and were more often on sick leave or unemployed, than cohabitating mothers (Table 1**).** After excluding mothers not cohabiting at 6 weeks postpartum (n = 71, out of which only 10 had responded to the 6 year postpartum survey), 112 (11%) were not cohabiting. These mothers displayed largely comparable background characteristics to the whole group included in the 6 year postpartum analyses (Table 1**).**

At 6 weeks postpartum, 778 (18.2%) of the mothers reported positive depressive symptom screenings once, whereas 259 (6.1%) twice. Among the mothers who participated at 6 years postpartum, 187 (16.5%) reported positive depressive symptom screenings once, 62 (5.5%) twice, and 29 (2.6%) three times. These 29 mothers represent 10.4% of all mothers with depressive symptoms (n = 278) included in the 6 years postpartum analyses. When excluding mothers who already stated they were non-cohabiting at 6 weeks, 182 (16.2%) reported positive depressive symptom screenings once, 61 (5.5%) twice, and 28 (2.5%) three times.

Persons who had experienced PND symptoms had higher odds of not cohabiting both at 6 weeks postpartum and 6 years postpartum. The point estimates were increasing with the number of posititve depression screenings, with the highest being at 6 years postpartum in mothers with three positive depression screenings (OR 4.1 95% CI 1.7–9.5). All associations except for one positive depression screening and cohabitation status at 6 weeks postpartum, held statistical significance when adjusted for age and education. However, the effect size of the associations decreased, and statistical significance was lost in the fully adjusted models except for three positive screenings and cohabitation at 6 years postpartum (Table 2**).** History of depression, experience of stressful life events/violence, and unplanned pregnancy were strong contributing factors. The results remained largely comparable when excluding mothers already not cohabiting at 6 weeks postpartum but then as at the 6 weeks postpartum no association held its significance at the fully adjusted model.

**Table 2:** Association between the number of positive depression screenings and cohabitation at 6 weeks and 6 years postpartum presented as logistic regression-derived odds ratios with 95% confidence intervals.

	Cohabitating, n (%)	Not cohabiting, n (%)	Unadjusted model	Model 1	Model 2
			OR (95% CI)	OR (95% CI)	OR (95% CI)
***Cohabitation status at 6 weeks postpartum***					
Positive depression screenings during pregnancy until 6wpp					
*No positive screening*	3,262 (76%)	45 (63%)	ref	ref	ref
*One positive screening (antenatal or 6wpp)*	761 (18%)	17 (24%)	1.6 (0.9, 2.8)	1.6 (0.9, 2.9)	1.1 (0.6, 2.0)
*Two positive screenings (antenatal and at 6wpp)*	250 (6%)	9 (13%)	**2.6 (1.3, 5.4)**	**2.6 (1.3, 5.5)**	1.5 (0.7, 3.2)
***Cohabitation status at 6 years postpartum***					
Positive depression screenings at pregnancy and at 6wpp/6mpp					
*No positive screening*	779 (77%)	73 (60%)	ref	ref	ref
*One positive screening (antenatal or 6wpp or 6mpp)*	157 (16%)	29 (24%)	**2.0 (1.2, 3.1)**	**1.9 (1.2, 3.1)**	1.5 (0.9, 2.4)
*Two positive screenings (antenatal or 6wpp or 6mpp)*	51 (5%)	11 (9%)	**2.3 (1.1, 4.6)**	**2.2 (1.1, 4.4)**	1.4 (0.7, 3.0)
*Three positive screenings (antenatal and 6wpp and 6mpp)*	21 (2%)	8 (7%)	**4.1 (1.7, 9.5)**	**3.6 (1.5, 8.6)**	**2.4 (1.0, 6.0)**
***Cohabitation status at 6 years postpartum, excluding women not cohabiting at 6 weeks***					
Positive depression screenings at pregnancy and at 6wpp/6mpp					
*No positive screening*	779 (77%)	69 (62%)	ref	ref	ref
*One positive screening (antenatal or 6wpp or 6mpp)*	156 (15%)	26 (23%)	**1.9 (1.2, 3.0)**	**1.8 (1.1, 3.0)**	1.4 (0.9, 2.4)
*Two positive screenings (antenatal or 6wpp or 6mpp)*	51 (5%)	10 (9%)	**2.2 (1.1, 4.6)**	2.0 (1.0, 4.3)	1.4 (0.6, 2.9)
*Three positive screenings (antenatal and 6wpp and 6mpp)*	21 (2%)	7 (6%)	**3.8 (1.5, 9.2)**	**3.2 (1.3, 8.0)**	2.0 (0.8, 5.3)

OR = odds ratio, CI = confidence intervals, 6wpp = six weeks postpartum, 6mpp = six months postpartum, 6ypp = six years postpartum.

Model 1. adjusted for age, education, and 6 weeks postpartum parity.

Model 2. additionally adjusted for planned pregnancy, history of depression, stressful life events, and experienced violence in relationships.

## Discussion

In this study, we found that 11.0% of mothers who were cohabiting at 6 weeks postpartum were not cohabiting at 6 years postpartum. Further, we found a positive association between PND symptoms and not cohabitating at both 6 weeks and 6 years postpartum. A relationship between longer duration of exposure to PND (numbers of positive depression screenings) and not cohabitating was noted, but it did not hold in the fully adjusted models. A significant association in the fully adjusted models was found only for mothers with three positive depression screenings and not cohabiting at 6 years postpartum. However, this finding had a wide 95% CI, due to the small number of mothers in some comparison groups.

Although our sample size exceeds previous studies from Sweden [5,25], the stability of our findings remains unclear, as the association between PND and non-cohabitation did not hold across most adjusted models. This highlights the need for studies with even larger sample sizes to confirm our findings. Noteworthy, various risk factors, including planned pregnancy, history of depression, stressful life events, and experienced violence in relationships, seem to be related to the development of PND and in parallel partially account for parental separation. Consequently, in almost all models it was shown that when risk factors are accounted, PND is no longer significantly related with cohabitation status. Thus, there is a need for future investigations to delve more into such comprehensive models to elucidate the complexity of the phenomenon and establish suitable practical interventions.

The results of our study are in accordance with the study by Kerstis et al. [25]; they found that mothers with depressive symptoms at three months postpartum had a higher risk of parental separation than mothers without depressive symptoms. Importantly, the aforementioned association was statistically significant when employing the EPDS mean as the threshold, but not when using a clinical cut-off [34]. Widarsson et al. [5], found depressive symptoms at three months postpartum to be associated with parental separation at 9–11 years postpartum; however, in multivariate analyses, only spouse relationship problems were associated with parental separation. Again, this study differed methodologically from ours as the PND symptoms were not categorized based on a cut-off. Thus, even though it suggested that those who were separated had higher depressive symptoms scores, the mean EPDS score in the separated group was 7, which is below the clinical cut-off [34].

The proportion of mothers not cohabiting at 6 years postpartum (11.0%) was lower in our study compared to Kerstis et al. [25] (20%) at 6–8 years postpartum and Widarsson et al. [5] (29%) at 9–11 years postpartum, possibly due to longer period after childbirth addressed in these studies. Moreover, individuals in the BASIC study tend to be slightly older and more highly educated than the overall general population [29], which are protective factors against parental separation and divorce [2,4,5,25].

In Sweden, pregnant persons are screened for PND symptoms at child healthcare centers at around 6–8 weeks postpartum. If screened positive, the mothers are referred for further evaluation [35]. Thereafter, maternal mental health is no longer assessed on a routine basis within healthcare. Our study found that a significant number of mothers experiencing PND symptoms did not recover within six months, possibly due to not being identified by the healthcare system [36]. It is known that individuals suffering from PND often have persisting depressive symptoms [19], but no guidelines suggest longer follow-up, which might be needed. Given the potential link between PND and parental separation, strengthening mental health support systems to address long-term depression trajectories could help mitigate consequences for both the mothers and their families.

Parental separation is classified as an adverse childhood experience (ACE) by the World Health Organization, as it has been associated with long-term negative health outcomes [37]. Children of separated parents are more likely to be exposed to household tobacco use [38,39], have lower compliance with vaccination schedules, [38,40], and experience increased psychosomatic problems [41]. Further, parental separation has been linked to depression in adolescence [42], and adult depression, anxiety, suicide attempts, suicidal ideation, distress, alcohol, smoking and drug abuse [37]. However, negative effects on children’s well-being are not necessarily caused by the parental separation itself, but rather by related factors, such as conflicts between parents [43], economic hardships, changes in parenting practices, and diminished parental well-being [44]. Conversely, research indicates that when parents maintain a cooperative approach after separation, there are higher levels of family functioning and fewer issues observed in children, including both internalizing and externalizing problems [45,46]. In Sweden, the impact of parental divorce on children’s well-being varies widely, with shared residence arrangements considered beneficial [47].

Although dissolving a troubled marital relationship may be a positive solution for some families [48], the association between PND and not cohabitating, underscores the importance to prioritize the efforts to prevent PND, as both PND and parental separation may have negative and possibly additive impacts on the health of mothers and children. According to our results, experience of depressive episodes before pregnancy, stressful life events, and intimate partner violence, often encountered among divorcees/separated mothers, contribute to low mental well-being perinatally [49]. Moreover, unplanned pregnancies seem to influence the relationship between PND symptoms and cohabitation status, particularly in the early postpartum period. Future studies could focus on these factors to gain further insight into their confounding impact on the association between PND and parental separation. In fact, our study points to them as important confounding factors but also targets for intervention. Moreover, as proposed by the literature, paternal PND is of particular interest to the studied association and should be taken into consideration in future studies.

However, when interpreting the results of this study we cannot rule out the possibility of reverse causality. We need to address the possibility that an impending change in cohabitation status or marital/relationship conflicts that were not formalized at the time of the depression assessment may have caused the depression, leading to a circular causality in the association. Even though, to our knowledge, this association has not been assessed yet, it is crucial that risk factors that are related with relationship dissolution, such as lack of partner support, are also risk factors for manifesting PND symptoms [28]. Thus, the temporal order of those phenomena is difficult to be delineated and need to be further investigated in longitudinal studies with information on both variables at more time points.

### Strengths and limitations

Strengths of this study include the relatively large sample size, the multiple assessments of depression, and the combined source of self-reports and journal entry data to assess parental cohabitation. Another strength of this study is the use of the EPDS, a widely recognized and validated screening tool for PND symptoms [50]. Employing EPDS allows for comparability with previous studies and enhances the validity of our findings. Moreover, given the high prevalence of underdiagnosed PND, utilizing a standardized tool increases the likelihood of capturing depressive symptoms that might otherwise go unrecognized [36]. This study has some limitations. The two sources for cohabitation were discordant for some mothers; in such situations, self-reported data were used, which might have introduced misclassification of the exposure. However, for the majority of the mothers, the two sources for cohabitation were in accordance. Additionally, the operationalization of cohabitation status could have led to misclassifications, as the two measurements differ with one assessing cohabitation with the mother’s partner at 6 weeks postpartum and the other the cohabitation with the child’s father at 6 years postpartum, who may not necessarily be the same person. Also, we lack data on depression status at 6 years postpartum. However, depression with onset later than 6 months postpartum is often not considered PND, and such misclassification (classifying exposed as non-exposed) should attenuate the association. Further, while EPDS has high sensitivity and specificity, there is a risk of misclassification of PND, as EPDS should be considered a screening tool and not a diagnostic tool. Additionally, the number of mothers not cohabitating with their partner at both examined time points was vastly smaller in comparison to those who continued to cohabit, potentially influencing the statistical power of the analyses and their capacity to discern differences. Furthermore, this study may be subject to selection bias as mothers with depression, and mothers who change their cohabitation status, might not participate in the 6-year follow-up or complete the assessments, to the same extent as non-depressed/cohabiting mothers. There is a risk of residual confounding as there might be unmeasured confounders, such as the relationship satisfaction, the socioeconomic status of the mothers and the mental status of the partner, which might also significantly impact the association.

### Clinical implication

Our results suggest, if confirmed in future studies, that more resources should be allocated to mothers presenting with high EPDS scores in pregnancy or early postpartum to prevent prolonged depressive episodes.

## Conclusion

While PND symptoms were initially associated with not cohabiting at both 6 weeks and 6 years postpartum, most associations did not remain significant after full adjustment. However, the significant association between prolonged PND exposure (three positive depression screenings) and not cohabiting at 6 years postpartum, despite a wide confidence interval, suggests a potential long-term impact of PND on family stability, warranting further research.
